# LOWER COMMUNITY LIVABILITY IS ASSOCIATED WITH HIGHER DEMENTIA PREVALENCE AMONG WOMEN IN SOUTH CAROLINA COUNTIES

**DOI:** 10.1093/geroni/igae098.0046

**Published:** 2024-12-31

**Authors:** Dana Alhasan, Marilyn Wende, Toni Antonucci, Florence Dallo, Maggi Miller

**Affiliations:** University of North Carolina, Charlotte, Charlotte, North Carolina, United States; University of Florida, Gainesville, Florida, United States; University of Michigan, Ann Arbor, Michigan, United States; Oakland University, Rochester, Michigan, United States; University of South Carolina, Columbia, South Carolina, United States

## Abstract

Community livability may be associated with lower dementia prevalence through access to healthcare professionals, for example. Given the high rates of dementia among older adults with a disproportionate impact on women, there is a need to understand how community livability may buffer against dementia and mitigate gender disparities. Yet, few studies have considered community livability as an exposure. Therefore, our exploratory study determined county-level associations between community livability and dementia prevalence by gender in South Carolina (SC). We retrieved county-level dementia prevalent cases in 2016 among ≥50 years old (N=46) from the SC Alzheimer’s Disease Registry. Community livability data (categorized into tertiles) were obtained from the American Association of Retired Persons’ Livability Index in 2018, which included seven domains: housing, neighborhood, transportation, physical environment, healthcare access, social engagement, and economic as well as educational opportunity. We conducted OLS regression analysis adjusting for county-level rurality to estimate beta coefficients and p-values. The study included 48.6% women (n=622) and 51.4% men (n=656). In adjusted analyses, low vs. high livability was associated with higher dementia prevalence (B=0.38; p-value=0.31). Although not statistically significant, the findings are in the expected direction. Low vs. high livability was associated with higher dementia among women (B=0.11; p-value=0.69), but lower dementia among men (B= -0.78; p-value=0.07). Our novel findings regarding communities with higher livability being associated with lower dementia highlight the potential health benefits of enhancing the livability of communities and is a first step towards future research to better understand this understudied relationship.

